# Supplementary material for: Multi-task gradient boosting with multi-modal molecular representations for simultaneous prediction of drug clearance and volume of distribution
Source: PLoS One. 2026 Apr 29;21(4):e0348173. doi: 10.1371/journal.pone.0348173 (PMC13127974; doi:10.1371/journal.pone.0348173)
Supplement: Attachment — Submitted filename: Response to Reviewers.docx [file pone.0348173.s002.docx]

**Response to Review Reports on “PONE-D-25-68444”**

“Multi-Task Gradient Boosting with Multi-Modal Molecular Representations for Simultaneous Prediction of Drug Clearance and Volume of Distribution”

First, we would like to thank the review team for their constructive and insightful comments. We have worked diligently and addressed all of the review team’s comments, which helped us to further improve the manuscript.

Below, we provide our point-to-point detailed responses to your comments, which are written in italics. We also indicated changes made in the main manuscript by using colored text. We hope you will find the next version of the manuscript worthy of publication. We would, of course, be happy to address any remaining concerns.

**Response to Reviewer 1**

*Comments 1:* *The presentation quality is a serious weakness. Many figures use extremely small fonts that are difficult to read even when zoomed in, particularly the BorutaSHAP plots and scatter plots comparing single-task and multi-task models. Given that interpretability (via SHAP) is emphasized as a key contribution, the unreadable axis labels, legends, and annotations significantly undermine the significance of the findings. This is not a cosmetic issue: poor readability directly affects the readers' ability to assess the claims. All figures should be regenerated with substantially larger fonts, simplified legends, and clearer layouts. In addition, some figures appear redundant (e.g., multiple scatter plots across splits conveying similar information) and could be consolidated to improve clarity.*

Response 1: We sincerely appreciate this important feedback. We have thoroughly revised all figures in the manuscript with the following changes: (1) Font sizes for axis labels, tick labels, legends, and annotations will be substantially increased in the final accepted version to ensure readability at standard print size; we acknowledge that this revision could not be completed within the current submission timeline and commit to addressing it fully prior to final acceptance. (2) The BorutaSHAP importance plots now display only the top selected features per target with simplified legends and cleaner layouts; (3) Regarding the scatter plots, Figs 3 and 4 serve distinct diagnostic purposes—Fig 3 presents train/validation/test scatter plots for the single-task LightGBM model, while Fig 4 presents the corresponding plots for the MTGBM model. These figures are necessary to assess each model's generalization behavior (e.g., overfitting) independently and are therefore retained as separate figures. To address the concern about redundancy, Fig 6 has been revised to serve as the primary comparative summary, directly overlaying LightGBM and MTGBM predictions on the test set alongside MSE comparison bars and residual analyses, allowing readers to assess the key performance differences in a single consolidated view. These revisions ensure that the key results, especially the interpretability analysis via SHAP, can be assessed clearly by readers. Additionally, three new figures have been added to support the revised analyses: Figs 7 and 8 present per-run MSE differences between MTGBM and LightGBM across 10 repeated random splits for CL and VD respectively, visually confirming the directional consistency of MTGBM's improvements; Fig 5 presents the range-stratified GMFE comparison for VD prediction, supporting the mechanistic interpretation of the MSE/GMFE divergence discussed in Section 4.3.

*Comments 2:* *The claimed novelty of the work is overstated. Multi-task gradient boosting machines with shared tree structures are not new and have already been formally introduced and analyzed. Moreover, from a practical standpoint, multi-output or multi-task tree-based models are well supported in widely used ecosystems such as LightGBM and scikit-learn, either directly or through standard wrappers. While it is true that such approaches are less commonly applied in PK prediction, this constitutes an application-level novelty rather than a methodological one.*

Response 2: We thank the reviewer for this well-founded critique and fully agree with the assessment. Multi-task gradient boosting with shared tree structures was formally established by Ying et al. (2022), and multi-output tree-based models are already available in widely used frameworks such as LightGBM and scikit-learn. We have revised the Introduction accordingly to explicitly reflect this. Specifically, the fourth paragraph now frames the MTGBM framework as building directly on the MT-GBM formulation of Ying et al. (2022), and replaces language such as "we present a novel framework" with "we propose and evaluate" to avoid overstating the methodological contribution. The phrase "novel" has been removed. The three primary contributions are now explicitly labeled as application-level: (1) systematic evaluation of combining multi-modal representations within a multi-task gradient boosting framework for PK prediction, (2) rigorous empirical evaluation using BorutaSHAP feature selection and repeated random split analysis, and (3) SHAP-based interpretability analysis. The closing sentence of the Introduction has similarly been revised from "our findings demonstrate consistent improvements" to "our results serve as a proof-of-concept," consistent with the reframing throughout the manuscript. Section 2.3 (Research Gaps and Our Contribution) has also been revised to state clearly that this work represents an application-level contribution rather than a methodological advance.

*Comments 3:* *Similarly, the construction of 'multi-task gradient boosting trees' in this manuscript largely follows established paradigms and does not introduce fundamentally new optimization strategies, regularization schemes, or task-coupling mechanisms. The improvements reported are modest (4.9% MSE reduction for CL and 10.8% for Vd) and, importantly, not statistically significant under the Diebold–Mariano test. As such, the authors should significantly temper their novelty claims and reframe the contribution as a careful application and validation study rather than a methodological advance.*

Response 3: We agree entirely with this assessment. The revised manuscript now consistently frames this work as a proof-of-concept application and validation study rather than a methodological advance. In the abstract, we now explicitly state that “the MTGBM showed modest improvements over single-task models in MSE and R² metrics for both CL and Vd predictions, though these improvements did not reach statistical significance.” The Discussion section (Section 5) similarly acknowledges that the 4.9% and 10.8% MSE reductions did not reach statistical significance under the Diebold–Mariano test, and frames these findings as “preliminary evidence suggesting the feasibility of the multi-modal, multi-task approach.” To further contextualize the robustness of the observed trends, we added a repeated random split analysis (10 independent splits; Section 3.6 and Section 4.4), in which MTGBM achieved lower MSE than LightGBM in 7 out of 10 runs for CL (win rate: 70%) and 8 out of 10 runs for VD (win rate: 80%), supporting a consistent directional tendency despite the lack of formal statistical significance.

*Comments 4:* *A major omission is the lack of discussion and comparison with recent blind-prediction challenges and benchmarks that strongly support the superiority of multi-modal, multi-task pretraining regimes. For example, recent work reported in Journal of Chemical Information and Modeling 65 (19), 10465-10476 demonstrates that large-scale, multi-modal, multi-task pretraining consistently outperforms task-specific models on ADME-relevant endpoints, especially in low-data regimes. Conceptually, the current MTGBM framework addresses a similar motivation but does so in a comparatively shallow, non-pretrained setting.*

Response 4: We thank the reviewer for highlighting this important body of literature. We have added a dedicated paragraph in Section 2.2 discussing large-scale pretraining and foundation model approaches. Specifically, we now cite Bang et al. (2025), who demonstrated substantial performance gains from multi-task pretraining across 21 ADME endpoints, and Cai et al. (2025) who introduced ChemFM, a 3-billion-parameter foundation model achieving consistent improvements across 34 molecular property prediction benchmarks. We explicitly position our MTGBM framework in relation to these pretraining-based approaches: while the pretraining paradigm leverages millions of molecules and substantial computational resources, our framework operates in a low-data regime (~700 compounds) where task-specific supervised embeddings offer a practical alternative. We acknowledge that our approach is methodologically shallower and non-pretrained, and discuss this distinction in both Section 2.3 and the Discussion (Section 5).

*Comments 5:* *The authors should explicitly discuss how their approach relates to, differs from, and potentially complements such pretraining-based frameworks. In particular, it would be valuable to clarify whether the modest gains observed here stem from limited dataset size (~700 compounds) and whether the approach would scale or remain competitive in settings where pretrained representations are available.*

Response 5: We have addressed this directly in both Section 2.3 and the Discussion (Section 5). In Section 2.3, we now state: “Unlike large-scale pretraining approaches that require millions of molecules and substantial computational resources, our framework operates in a constrained low-data setting (~700 compounds) where task-specific supervised embeddings offer a practical alternative. While foundation model-based representations may yield stronger generalization, exploring whether the MTGBM multi-task boosting layer can serve as a lightweight downstream learner on top of such pretrained embeddings remains a promising direction for future work.” In the Discussion, we note that the limited dataset size likely constrains statistical power and discuss that the MTGBM framework may demonstrate stronger or more statistically reliable improvements when applied to larger datasets or when paired with more generalizable pretrained representations. This is now listed as an explicit future research direction.

*Comments 6:* *Relatedly, the manuscript does not engage with the rapidly growing body of work on large models and foundation models for molecular and drug discovery. Recent studies (e.g., arXiv:2511.11257) have demonstrated that large, pretrained models (often trained across millions of molecules and multiple objectives) can achieve outstanding predictive power on downstream PK and ADME tasks, frequently surpassing classical ML pipelines even without extensive feature engineering.*

Response 6: We have incorporated discussion of foundation models for molecular property prediction in Section 2.2. We now cite relevant recent work including Cai et al. (2025) (ChemFM) and Bang et al. (2025), and acknowledge that such large pretrained models frequently outperform classical ML pipelines on ADME tasks. In the Discussion (Section 5), we explicitly note that while our current approach relies on relatively compact CNN and MLP embeddings trained on ~700 compounds, the broader landscape is moving toward foundation model-scale representations. We discuss that the MTGBM multi-task boosting framework could potentially serve as a lightweight downstream learner on top of such foundation model embeddings, representing a promising direction for future work. This framing positions our study as a proof-of-concept that motivates such integration rather than claiming competitiveness with foundation model approaches.

*Comments7:* *Given this context, the authors' reliance on relatively small CNN and MLP embeddings trained specifically for CL and Vd should be critically discussed. Are the gains from MTGBM expected to persist when stronger, pretrained representations are used? Could the proposed multi-task boosting layer act as a lightweight downstream learner on top of foundation-model embeddings? Without such discussion, the work risks appearing disconnected from the current trajectory of the field.*

Response 7: We have directly addressed both questions raised by the reviewer. In the Discussion (Section 5), we now include the following: (1) Regarding persistence of gains with stronger representations—we discuss that given the MTGBM framework’s consistent directional improvements even with small-scale task-specific embeddings, it is plausible that stronger pretrained representations would further amplify these gains, particularly for capturing general chemical knowledge beyond the training distribution. (2) Regarding the multi-task boosting layer as a lightweight downstream learner—we explicitly propose this as a future research direction, noting that the MTGBM framework’s modular architecture (where embeddings are extracted independently before being fed into the gradient boosting model) naturally lends itself to substitution of task-specific embeddings with pretrained foundation model representations. These additions ensure that the work is appropriately connected to the current trajectory of the field.

**Response to Reviewer 2**

*Comments 1:* *However, I do have some concerns about the work that should be addressed before publication. The most significant issue is the lack of statistical significance in the improvements over single-task models. While the authors acknowledge this limitation, they somewhat overstate the potential of their approach throughout the manuscript. The Diebold-Mariano test results with p-values of 0.117 and 0.245 for CL and VD respectively indicate that we cannot rule out the possibility that the observed improvements occurred by chance. Given that the dataset contains only about 700 compounds, this is perhaps not surprising, but the discussion should more carefully frame these findings as preliminary evidence rather than definitive proof of the method's superiority.*

Response 1: We thank the reviewer for this important methodological point. We have revised the manuscript to more carefully frame our findings as preliminary evidence throughout. The abstract now explicitly states that improvements “did not reach statistical significance” and characterizes the results as suggesting “the potential” rather than establishing superiority of the multi-task approach. In the Discussion (Section 5), we explicitly acknowledge the p-values of 0.117 (CL) and 0.245 (VD) from the Diebold–Mariano test and discuss that the limited dataset size (~700 compounds) likely constrains statistical power. We have also added a repeated random split analysis (10 independent splits, Section 3.6 and Section 4.4) showing that MTGBM achieved lower MSE in 7/10 runs for CL and 8/10 runs for VD, which provides complementary evidence of a consistent directional tendency without overstating statistical conclusions. Language throughout the manuscript has been revised to replace definitive claims with appropriately hedged expressions.

*Comments 2:* *The dataset size itself poses another challenge. With only around 700 compounds split into training, validation, and test sets, the test set ends up being quite small at 139 samples. This raises questions about the stability of the performance metrics and whether they might be heavily influenced by a few outliers. The authors mention using a robust optimization algorithm for splitting, but it would be helpful to know more about how stable the results were across different random splits.*

Response 2: We agree that the small test set (139 samples) raises legitimate concerns about metric stability. To address this, we have added a repeated random split analysis (Section 3.6 and Section 4.4) in which the complete modeling pipeline was executed across 10 independent random seeds, each generating a new 60/20/20 train/validation/test split. Results are reported as mean ± standard deviation across all 10 runs (Table 2). For CL prediction, MTGBM achieved mean MSE of 16.663 ± 3.326 versus 17.765 ± 3.038 for LightGBM, with MTGBM outperforming in 7 of 10 runs. For VD prediction, MTGBM achieved mean MSE of 0.669 ± 0.143 versus 0.766 ± 0.185, with MTGBM outperforming in 8 of 10 runs. We also noted and discuss the slightly higher standard deviation of MTGBM in CL predictions, which we attribute to the use of estimated rather than fully optimized hyperparameters. These results confirm that the primary single-split findings reflect a consistent directional tendency rather than an artifact of a particularly favorable partition.

*Comments 3:* *I also have some methodological concerns about the feature extraction process. The authors trained separate CNN and MLP models for CL and VD predictions before extracting embeddings for the MTGBM model. This creates a potential information leakage issue since these embedding models were trained using the same data that later gets used for training and evaluating the MTGBM. Ideally, the embedding extraction should be done in a way that keeps test data completely separate, perhaps through nested cross-validation or by training the embedding models only on training data before any validation or testing occurs.*

Response 3: We thank the reviewer for raising this critical methodological point. We have carefully reviewed our pipeline and clarified the embedding extraction protocol in Sections 3.3.1 and 3.3.2 of the revised manuscript. Both CNN and MLP embedding models were trained under a strict three-step protocol: (1) hyperparameter optimization using Optuna (100 trials) was performed by evaluating candidate configurations solely on the validation set—the test set was not involved at this stage; (2) final model training used the combined train+validation data with best hyperparameters and early stopping, without accessing the test set; (3) embeddings were extracted from the trained model in inference mode, applied independently to each data split, with no further parameter updates. Critically, the test set was reserved exclusively for final embedding extraction and was never used in any model fitting or hyperparameter selection step. To avoid redundancy, the test set separation description in Section 3.6 has been condensed to a brief cross-reference to Sections 3.3.1 and 3.3.2, where the full protocol is described. We acknowledge that this design, where the embedding models are trained on the same target variables used for final prediction, may still create a form of target-informed feature encoding, which we discuss as a limitation in Section 5. A fully clean alternative would require unsupervised molecular embeddings, which we identify as a direction for future work.

*Comments 4:* *The hyperparameter optimization approach for the MTGBM model is another area that needs clarification. The authors mention using "estimated optimal parameters" from single-task models due to computational constraints. While this is understandable, it does mean we cannot be sure that the MTGBM model is performing at its full potential. A properly optimized multi-task model might show larger improvements, or conversely, the current results might be underestimating its true performance. Either way, this limitation should be more prominently discussed.*

Response 4: We agree that this is an important limitation that deserved more prominent treatment. In the revised manuscript, we have expanded the limitations discussion in Section 5 to explicitly address both directions of uncertainty: the current results may underestimate MTGBM’s true potential if the optimal multi-task hyperparameters differ substantially from single-task estimates, or they may overestimate it if the estimated parameters happen to favor the training data. We also note in Section 4.4 that the slightly higher standard deviation of MTGBM’s CL MSE across 10 repeated random splits (3.326 vs 3.038 for LightGBM) is consistent with greater performance variability in the absence of dedicated multi-task hyperparameter optimization. The Methods section (Section 3.5.2) now more clearly explains why dedicated multi-task hyperparameter optimization was not performed—the custom multi-task objective function requires substantially higher computational cost than single-task LightGBM—and we identify dedicated MTGBM hyperparameter optimization as a priority for future work.

*Comments 5:* *The interpretability analysis using SHAP values raises some questions about what exactly the MLP and CNN embeddings represent. These are learned representations from neural networks trained specifically to predict CL and VD, so it is somewhat circular when they emerge as important features in the SHAP analysis. The authors interpret this as evidence of successful cross-task information sharing, but it might simply reflect that these embeddings already contain target-specific information from their training process. A cleaner approach might have been to use unsupervised molecular embeddings that were not trained on the prediction tasks themselves.*

Response 5: We thank the reviewer for this insightful point. We have revised the interpretability analysis (Section 4.5) and Discussion (Section 5) to explicitly acknowledge this limitation. We now state: “Since the CNN and MLP embeddings were trained directly on CL and VD as target variables, their prominence in SHAP analysis may partly reflect their target-specific supervised training rather than solely the effect of multi-task learning.” We note that the cross-task feature prominence (e.g., mlp_vec1_VD ranking among top CL predictors) is consistent with cross-target information sharing, but cannot be cleanly separated from the target-encoding property of the supervised embeddings. We agree that unsupervised molecular embeddings (e.g., from self-supervised pretraining methods) would provide a cleaner baseline for such interpretability analysis, and we explicitly identify this as a direction for future work. The current SHAP analysis is now framed as providing exploratory insights into feature importance patterns rather than definitive evidence of cross-task generalization.

*Comments 6:* *I noticed some inconsistencies in the reported results that need attention. For VD prediction, the MSE improved substantially from 0.950 to 0.848, yet the GMFE increased from 1.844 to 1.938. The authors suggest this indicates the importance of evaluating multiple metrics, which is valid, but this pattern deserves more discussion. Typically, improvements in MSE would be expected to correspond with better GMFE, so the divergence here might indicate something interesting about the error distribution that could be explored further.*

Response 6: We thank the reviewer for prompting a deeper investigation into this divergence. To address this, we conducted a range-stratified analysis of VD prediction (Table 3 in the revised manuscript, Section 4.3), partitioning test compounds into three ranges: low (<0.5 L/kg, n=45), mid (0.5–2.0 L/kg, n=49), and high (>2.0 L/kg, n=23). The analysis revealed that MTGBM demonstrated superior GMFE in both the mid (GMFE: 1.455 vs 1.500, Δ = −0.045) and high (GMFE: 2.332 vs 2.389, Δ = −0.057) VD ranges. However, in the low VD range (<0.5 L/kg), MTGBM showed substantially higher GMFE (2.409 vs 2.022, Δ = +0.387), which drove the overall GMFE deterioration. We discuss in Section 5 that GMFE is particularly sensitive to relative fold errors in the low VD range, and that MTGBM’s shared tree optimization may introduce a trade-off that compromises prediction precision for low-value compounds. This pattern is consistent with a distributional shift in residuals concentrated in the low VD range rather than uniform performance degradation across the full distribution, and we now explicitly discuss this mechanistic interpretation in the manuscript.

*Comments 7:* *The manuscript could also benefit from more careful editing in places. The literature review section repeats some content between sections 2.1 and 2.2, and there are minor formatting inconsistencies in the references. The figures mentioned in the text, particularly Figures 1 through 6, are referenced but not actually included in the manuscript file, making it impossible to fully evaluate the visual presentations of the results.*

Response 7: We apologize for these presentation issues. In the revised manuscript: (1) The repeated content between Sections 2.1 and 2.2 has been identified and removed—specifically, the sentence regarding Li et al. (2024) and hybrid ML-PBPK approaches was present in both sections and has been retained only in Section 2.1 where it contextually belongs; (2) Reference formatting has been reviewed and standardized throughout; (3) All figures (Figs 1–9) are now included in the revised manuscript submission as embedded images. Figs 1–6 correspond to the original analyses (BorutaSHAP selection plots, scatter plots, comparative performance summary, and SHAP interpretability). Figs 7 and 8 are newly added figures presenting per-run MSE differences across 10 repeated random splits for CL and VD respectively, and Fig 5 is a newly added range-stratified GMFE comparison for VD prediction. We have ensured that all figure numbers cited in the text correspond correctly to the attached figures.

*Comments 8:* *Despite these concerns, the work makes a valuable contribution by demonstrating how multi-task learning can be applied to pharmacokinetic prediction. The idea of leveraging correlations between related parameters is conceptually sound and worth exploring further. The authors have been transparent about their limitations, which is commendable. With some revisions to address the methodological issues and a more nuanced interpretation of the results, this could become a useful addition to the literature on computational pharmacokinetics. The framework they have developed could potentially be extended to other ADME properties, and their careful documentation of the modeling process provides a good foundation for others building on this work.*

Response 8: We sincerely appreciate the reviewer’s encouraging assessment and constructive perspective. We have worked to address all methodological concerns raised, strengthen the discussion of limitations, and more carefully calibrate the interpretation of results throughout the manuscript. We believe that the additions—including the repeated random split analysis, range-stratified GMFE analysis, clarified embedding protocol, and expanded discussion of pretraining-based approaches—substantially improve the rigor and nuance of the work. We hope the revised manuscript now meets the standard for publication in *PLOS One*.

We are grateful to both reviewers for their thorough and constructive engagement with our work. All changes have been marked in the revised manuscript using colored/tracked text for easy identification.
